# A Manuel of Pathology

**Published:** 1876-06

**Authors:** 


### Books Reviewed.

*A Manual of Pathology. For the use of Students and Practitioners of Medicine.* By ERNST WAGNER, Professor of General Pathology and Pathological Anatomy in the University of Leipzig, etc. Translated from the sixth German edition, by JOHN VAN DUYN, A. M., M. D., and E. C. SEGUIN, M. D. New York: Wm. Wood & Co., 1876. Buffalo: H. H. Otis.

The first edition of this work was published in German in 1862, under the title Uhle and Wagner's Manual of General Pathology, the work being edited by Wagner.

Of the present edition, the American editor says: "No book in the English language gives such a thorough *resume* of the elements of medicine."

In the first portion of the work are articles upon the general conception and forms of morbid states, nature and extension of disease, its symptoms, prognosis, duration, course and termination by death and cure, agony, apparent death, and causes of death.

In part second, on general etiology, the authors treat of—1st, internal causes, inheritance, age, sex, constitution, etc. 2d, external causes, as atmospheric influences, soil, climate, dwelling, clothing, food, drink, occupation, parasites, contagions and miasmata. A careful consideration of the article on parasites, which includes fungi bacteria, etc., will place the reader in a position better to understand the numerous theories and writings upon these subjects.

The third and fourth parts comprise the balance of the work. Part Third is upon general pathological anatomy and physiology, and is divided into three sections—1. Local disturbances of the circulation. 2. Inflammation. 3. General disturbances of nutrition. Under the last section are included retrograde metamorphoses—fatty, lardaceous culloid, pigmentary, etc., and progressive metamorphoses—regeneration, hypertrophy and new formations or morbid growths. The portion on morbid growths occupies a large portion of the work, over one hundred pages, and is one of the most interesting sections. The author follows the classification of Virchow.

Part Fourth is a consideration of the Pathology of the Blood, including anomalies of the size and shape of the blood-corpuscles, anæmia, acute and chronic, including chlorosis and Addison's disease; changes in the amount of hænia-globuline, of albumen, and of water in the blood; lencocythæmia, pyæmia, diabetes, fever, marasmus, etc.

The amount of labor spent in the preparation of this volume must have been simply enormous. Every chapter is opened with an extended reference to works upon the subjects of which it treats, and throughout the entire text a large number of authorities are referred to. The work is one which will amply repay perusal, in fact, no physician should neglect to read it, and the student should make it one of his most cherished text-books.

